# *Lactobacillus paracasei* KW3110 Prevents Blue Light-Induced Inflammation and Degeneration in the Retina

**DOI:** 10.3390/nu10121991

**Published:** 2018-12-15

**Authors:** Yuji Morita, Yukihiro Miwa, Kenta Jounai, Daisuke Fujiwara, Toshihide Kurihara, Osamu Kanauchi

**Affiliations:** 1 Research Laboratories for Health Science & Food Technologies, Kirin Company, Ltd., 1-13-5, Fukuura Kanazawa-ku, Yokohama-shi 236-0004, Japan; d-fujiwara@kirin.co.jp (D.F.); kanauchio@kirin.co.jp (O.K.); 2 Laboratory of Photobiology, Keio University School of Medicine, Tokyo 160-8582, Japan; yukihiro226@gmail.com (Y.M.); kurihara@z8.keio.jp (T.K.); 3 Department of Ophthalmology, Keio University School of Medicine, Tokyo 160-8582, Japan; 4 Technical Development Center, Koiwai Dairy Products Co Ltd. Sayama, Saitama 350-1321, Japan; k_jounai@koiwai-dairy.co.jp

**Keywords:** *Lactobacillus paracasei* KW3110, retina, light, macrophage

## Abstract

Age-related macular degeneration and retinitis pigmentosa are leading causes of blindness and share a pathological feature, which is photoreceptor degeneration. To date, the lack of a potential treatment to prevent such diseases has raised great concern. Photoreceptor degeneration can be accelerated by excessive light exposure via an inflammatory response; therefore, anti-inflammatory agents would be candidates to prevent the progress of photoreceptor degeneration. We previously reported that a lactic acid bacterium, *Lactobacillus paracasei* KW3110 (*L. paracasei* KW3110), activated macrophages suppressing inflammation in mice and humans. Recently, we also showed that intake of *L. paracasei* KW3110 could mitigate visual display terminal (VDT) load-induced ocular disorders in humans. However, the biological mechanism of *L. paracasei* KW3110 to retain visual function remains unclear. In this study, we found that *L. paracasei* KW3110 activated M2 macrophages inducing anti-inflammatory cytokine interleukin-10 (IL-10) production in vitro using bone marrow-derived M2 macrophages. We also show that IL-10 gene expression was significantly increased in the intestinal immune tissues 6 h after oral administration of *L. paracasei* KW3110 in vivo. Furthermore, we demonstrated that intake of *L. paracasei* KW3110 suppressed inflammation and photoreceptor degeneration in a murine model of light-induced retinopathy. These results suggest that *L. paracasei* KW3110 may have a preventive effect against degrative retinal diseases.

## 1. Introduction

In recent years, blue light has been used in several visual display terminals (VDTs), including computers, smart phones, and tablet devices; thus, opportunities of human exposure to blue light have increased. Excessive exposure to blue light can cause photoreceptor degeneration in the retina [1] and may be related to age-related macular degeneration (AMD) [2,3] and retinitis pigmentosa [4]. AMD and retinitis pigmentosa are the leading causes of blindness in the elderly population [5]. Recently, natural compounds in foods have attracted worldwide attention in an attempt to treat light-induced ocular problems, in particular, antioxidants in foods [6,7,8]. However, the mechanism of light-induced retinal damage has not been completely elucidated.

Although multiple factors such as oxidative stress and hypoxia have been reported to have a critical role in photoreceptor degeneration [9], retinal inflammation is also believed to be associated with the progression of photoreceptor degeneration [10,11,12]. In a previous report, recruitment and polarization of macrophages were shown to be involved in the pathogenesis of light-induced retinal degeneration in vivo [10]. Macrophages can be grouped into at least two subgroups, the classically activated inflammatory M1 phenotype and the alternatively activated M2 phenotype [13,14]. M1 macrophages produce several inflammatory cytokines including interleukin-1β (IL-1β) and cause inflammatory reactions. In contrast, M2 macrophages are associated with anti-inflammatory reactions including tissue remodeling through the production of anti-inflammatory cytokines such as interleukin-10 (IL-10) [15,16]. In the retina, the polarization of M2 macrophages is also thought to promote retinal cell survival in several mouse models [10,17].

Dietary nutrients or constituents have been reported to have potential in protecting photo-stressed retina [18,19]. However, the mechanism of action especially related to retinal inflammation is not well understood. Lactic acid bacteria have been widely used as sources of probiotics and paraprobiotics to enhance gut barrier function and improve the immune system. Some strains have been reported to attenuate several inflammatory phenomena including diarrhea, allergies, and metabolic disorders.

Our group has previously reported that *Lactobacillus paracasei* KW3110 (*L. paracasei* KW3110) suppressed excessive inflammation including dermatitis in mice [20,21,22] and humans [23]. In addition, we have shown that the intake of *L. paracasei* KW3110 mitigated VDT load-induced ocular disorders, including eye fatigue, in Japanese healthy adults [24]. In this study, we have investigated the ability of *L. paracasei* KW3110 to activate M2 macrophages in vitro and in vivo to attenuate blue light-induced retinal degeneration. We then examined the protective effects of *L. paracasei* KW3110 on retinal functions.

## 2. Materials and Methods

### 2.1. Animals

Four-week-old mice (BALB/c, male) were purchased (Charles River Japan, Kanagawa, Japan) and acclimatized for 1 week with free access to water and a basic diet AIN93G (Oriental Yeast, Tokyo, Japan) before all experiments were performed.

All animal procedures and experiments were performed in accordance with the Association for Research in Vision and Ophthalmology Statement for the Use of Animals in Ophthalmic and Vision Research. Animal procedures and experiments were also approved by the Laboratory Animal Care Committee for Experimental Animals of our institute: the approval ID was AN10134-Z00. All efforts were made to minimize animal suffering.

### 2.2. Preparation of Bone Marrow-Derived Macrophages

Bone marrow-derived M2 macrophages were generated as previously described [25,26]. Briefly, bone marrow cells were extracted from BALB/c mice, and erythrocytes were generated and harvested after brief exposure to 0.168 M NH_4_Cl. Cells were then cultured at a density of 5 × 10^5^ cells/mL for 7 days in RPMI1640 medium supplemented with 10% fetal calf serum (FCS) and 5,000 U/mL of macrophage colony stimulating-factor (M-CSF; R and D Systems, Minneapolis, MN, USA). Lipo-teichoic acid (LTA; Invitrogen, Carlsbad, CA, USA) was added at a concentration of 10 ng/mL and *L. paracasei* KW3110 was added at concentrations of 0.1, 1, and 10 µg/mL. The cultures were continued for 24 h. *L. paracasei* KW3110 was prepared as described in a previous study [23].

### 2.3. Enzyme-Linked Immunosorbent Assay (ELISA)

The concentrations of cytokines in cell culture supernatants were measured using a mouse IL-10 ELISA kit (BD Biosciences, San Jose, CA, USA).

### 2.4. Oral Administration and Sample Collection

The mice were orally administered saline (Otsuka Pharmaceutical) containing 50 mg of heat-killed *L. paracasei* KW3110. Mesenteric lymph nodes (MLNs) were removed at 0, 2, 6, 10, and 24 h after treatments (BALB/c, male, *n* = 8/each time point). The tissues were soaked in RNAlater RNA Stabilization Reagent (Qiagen, Hilden, Germany) and kept at −80 °C until RNA extraction.

### 2.5. RNA Preparation and Quantitative RT-PCR from Tissues

Total RNA was extracted from MLNs using the RNeasy Mini kit (Qiagen), and cDNAs were prepared using an iScript cDNA synthesis kit (BioRad, Hercules, CA, USA) according to the manufacturer’s instructions. The resulting products were subjected to quantitative RT-PCR using SYBR Premix Ex Taq (Takara Bio, Otsu, Japan) and a LightCycler PCR system (Roche Diagnostics, Basel, Switzerland). The relative expression levels of the gene were normalized to glyceraldehyde-3-phosphate dehydrogenase (*Gapdh*). The primers used for PCR were as follows: *Gapdh* forward (F) (AACGACCCCTTCATTGAC) and *Gapdh* reverse (R) (TCCACGACATACTCAGCAC), *Il10* F (CAGAGCCACATGCTCCTAGA) and *Il10* R (TGTCCAGCTGGTCCTTTGTT).

### 2.6. Light Exposure

After acclimatization, the mice (BALB/c, male) were divided by equal average weights into three groups (*n* = 6). The non-light exposure control mice group and the light exposure mice group were maintained on AIN93G purified rodent diet (Zeigler, Gardners, PA, USA). In addition, the light exposure *L. paracasei* KW3110 mice group was fed the AIN93G diet containing approximately 1 mg heat-killed *L. paracasei* KW3110/day/mouse. All mice were housed in specific pathogen-free conditions under a 12-h light-dark photo cycle and had ad libitum access to water and the diet. Two weeks later, light exposure experiments were performed. Mice were exposed to blue light as previously described with slight modifications [18]. Briefly, the mice were dark-adapted for 12 h before light exposure. The mice were then exposed to 5000 lux of blue light (CCS Inc., Kyoto, Japan, peak at 470 nm) for 3 h, starting at 9:00 a.m., in exposure boxes maintained at 23 °C. After light exposure, the mice were maintained under a dim cyclic light (5 lux, 12 h on/off).

### 2.7. Retinal Cell Preparations

Three days after the start of light exposure, the retinas were digested with 1 mg/mL collagenase II (Worthington, Lakewood, NJ, USA) for 40 min at 37 °C in Hank’s Balanced Salt Solution (HBSS) buffer with 1.0% bovine serum albumin (BSA). The tissue digest was then filtered through a 70 μm cell strainer and washed with HBSS buffer with 1.0% BSA for 5 min at 1300 rpm and at 4 °C. The supernatant was carefully removed and the digested tissue pellet was resuspended to form a single-cell suspension.

### 2.8. Flow Cytometry Analyses

The retinal cells were stained with fluorescent dyes conjugated to antibodies as follows: CD206-FITC (C068C2; BioLegend, San Diego, CA, USA); 7-AAD (BD Pharmingen, San Jose, CA, USA); CD11b-APC-Cy7 (M1/70; BD Biosciences San Jose, CA, USA); f4/80-PE-Cy7 (BM8; BioLegend). After staining, the cells were washed twice with a FACS buffer (0.5% BSA in PBS buffer) and suspended in the FACS buffer for FACS analyses. Data were collected using a FACS Canto II flow cytometer (BD Biosciences) and analyzed by FCS Express software (De Novo Software, Los Angeles, CA, USA). The 7-AAD− CD11b+ f4/80+ cells were defined as retinal macrophages.

To investigate intracellular cytokine production, retinal cells were treated with a leukocyte activation cocktail with BD GolgiPlug^TM^ (BD Biosciences) for 4.5 h and with a BD Cytofix/Cytoperm Fixation/Permeabilization^TM^ kit (BD Biosciences) and then stained with the following antibodies: TNF-α-FITC (MP6-XT22; eBiosciences, San Diego, CA, USA); IL-10-PE (GK1.5; BioLegend); CD11b-APC-Cy7 (M1/70; BD Biosciences); F4/80-PE-Cy7 (BM8; BioLegend); and 7-AAD (BD Pharmingen, San Jose, CA, USA). The 7-AAD− CD11b+ f4/80+ cells were defined as retinal macrophages. Data were collected using a FACS Canto II flow cytometer (BD Biosciences) and analyzed by FCS Express software (De Novo Software).

### 2.9. Analysis of Cytokine Concentrations

The retinal cells were cultured for 24 h in RPMI 1640 medium supplemented with 10% FCS to evaluate the production of inflammatory cytokines. Supernatants were collected and analyzed for cytokine concentrations using a Bio-Plex Pro mouse cytokine assay kit (Bio-Rad).

### 2.10. Measurements of the Retinal Thickness

One week after the start of light exposure, eye balls were fixed in neutral 10% formalin and decalcified. The tissues were sectioned including the regions from the optic nerve head to the most peripheral, then stained with hematoxylin and eosin. The outer nuclear layer (ONL) thickness in the retinal section was measured in all areas. We randomly selected ten observation points in each image and averaged using WinROOF software (MITANI Corporation).

### 2.11. Electroretinography (ERG)

After acclimatization, the mice (BALB/c, male) were divided by equal average weights into two groups. The control mice group (*n* = 4) was fed AIN93G diets. The *L. paracasei* KW3110 mice group (*n* = 4) was fed AIN93G containing approximately 1 mg heat-killed *L. paracasei* KW3110/day/mouse. All mice were housed in specific pathogen-free conditions under a 12-h light-dark (about 700 lux) photo cycle and had ad libitum access to water and the diet. Two weeks later, the mice were dark-adapted for 12 h and then placed under dim red illumination before conducting ERGs. The mice were anesthetized with an MMB combination anesthetic containing midazolam (4 mg/kg, SANDOZ, Yamagata, Japan), medetomidine (0.75 mg/kg, Nippon Zenyaku Kogyo Co., Ltd., Fukushima, Japan) and butorphanol tartrate (5 mg/kg, Meiji Seika Pharma, Tokyo, Japan) and placed on a heating pad to maintain their body temperatures at 35–36 °C throughout the experiments. The pupils were dilated with a single drop of a mixed solution of 0.5% tropicamide and 0.5% phenylephrine (Santen Pharmaceutical, Osaka, Japan). The ground and reference electrodes were then placed on the tail and subcutaneously between the eyes, respectively, while the active gold wire electrodes were placed on the cornea. The recordings were performed with a Ganzfeld dome, an acquisition system, and LED stimulators (PuREC, MAYO Corporation, Inazawa, Japan). The amplitude of the a-wave was measured from the baseline to the trough of the a-wave. The amplitude of the b-wave was determined from trough of the a-wave to the peak of the b-wave.

### 2.12. Statistical Analysis

**Figure 1:**
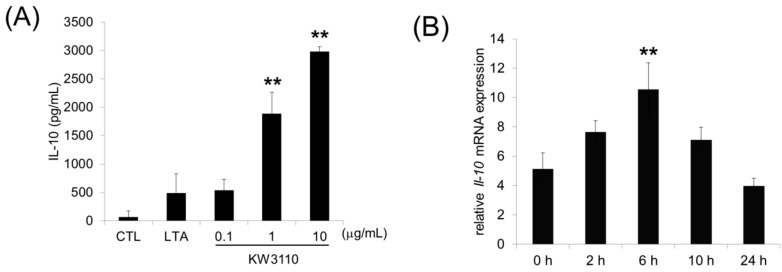
Effects of *L. paracasei* KW3110 on the activation of M2 macrophages and induction of IL-10 production. (**A**) Bone marrow-derived M2 macrophages were stimulated with lipo-teichoic acid (10 ng/mL), and *L. paracasei* KW3110 (0.1, 1, or 10 μg/mL) and the amounts of secreted IL-10 were measured by ELISA. (**B**) Relative *IL-10* mRNA expression was measured using PCR. Values are represented as the mean ± SEM. Significance was assumed if the *p* value was < 0.05. ** *p* < 0.01. LTA, lipo-teichoic acid treated group; CTL, control; KW3110, *L. paracasei* KW3110 treated group.

All values are presented as the mean ± SEM. Statistical differences for the results of Figure 1 were performed using Dunnett’s test for post-hoc comparisons. Statistical differences between three groups (control mice group fed a control diet without light exposure, light control mice group fed a control diet with light exposure, and *L. paracasei* KW3110 mice group fed a diet containing *L. paracasei* KW3110 with light exposure) were analyzed by one-way analysis of variance (ANOVA), followed by the Tukey-Kramer test with significance set at *p* < 0.05. Statistical differences between the two groups (light control mice group fed a control diet with light exposure and *L. paracasei* KW3110 mice group fed a diet containing *L. paracasei* KW3110 with light exposure) were determined using an unpaired, two-tailed Student’s *t*-test with significance set at p < 0.05. All statistical analyses were performed using the Ekuseru-Toukei 2012 software program (Social Survey Research Information, Tokyo, Japan).

## 3. Results

### 3.1. L. paracasei KW3110 Activates M2 Macrophages In Vitro and Induces IL-10 Production In Vivo

In order to determine the effects of *L. paracasei* KW3110 on M2 macrophage activation, bone marrow-derived M-CSF-induced M2 macrophages were treated with *L. paracasei* KW3110 and IL-10 levels, as a marker of M2-polarization [27], were measured in culture supernatants. *L. paracasei* KW3110 at 0.1–10 μg/mL induced IL-10 production in a concentration-dependent manner (Figure 1A). In the previous report, our team showed that orally provided *L. paracasei* KW3110 (50 mg/head) interacted with the immune cells in the gut [19]. To examine IL-10 induction of *L. paracasei* KW3110 in vivo, we evaluated IL-10 gene expression in mesenteric lymph nodes (MLNs) at several time points after oral administration of 50 mg/head *L. paracasei* KW3110 in mice. The *IL-10* mRNA level in MLNs significantly increased 6 h after oral administration and decreased to the basal level 24 h after administration (Figure 1B). These results suggest that *L. paracasei* KW3110 activated M2 macrophages inducing the production of IL-10.

### 3.2. L. paracasei KW3110 Induces Retinal M2 Macrophages Following Light Exposure

**Figure 2:**
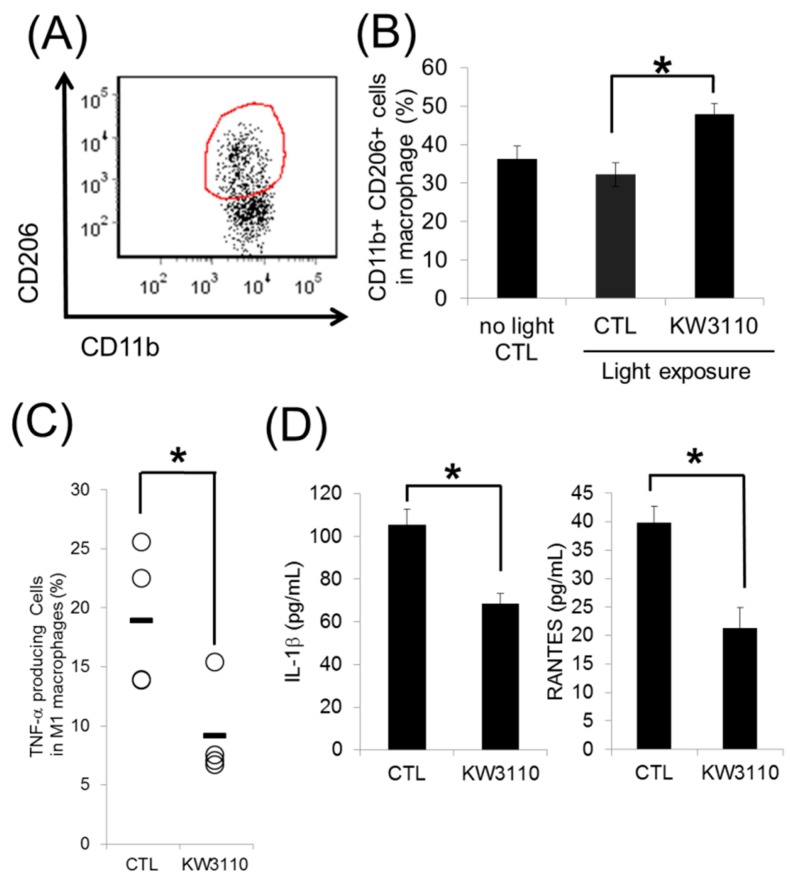
Effect of *L. paracasei* KW3110 on the induction of M2 macrophage in the retina of a light-exposed mouse model. (**A**) Representative flow cytometry data of CD11b-positive and CD206-positive cells from the blue light-exposed mice fed a diet containing *L. paracasei* KW3110 (KW3110) or fed a control diet (CTL). (**B**) The ratio of CD11b-positive, f4/80-positive, and CD206-positive M2 macrophage cells to CD11b-positive and f4/80-positive macrophage cells. (**C**) The ratio of TNF-α-producing cells in CD11b-positive M1 macrophage cells. To detect inflammatory cytokine-producing cells, retinal cells were cultured under stimulation with Leukocyte Activation Cocktail plus BD GolgiPlug™ and analyzed by flow cytometry. (**D**) The production of inflammatory cytokines IL-1β and RANTES in the retinal cell culture medium. CTL, control; KW3110, *L. paracasei* KW3110; TNF-α, tumor necrosis factor-α; IL-1β, interleukin-1β; RANTES, regulated on activation, normal T cell expressed and secreted. Values are presented as the mean ± SEM. Significance was assumed if the *p* value was < 0.05. * *p* < 0.05.

We next investigated the effects of *L. paracasei* KW3110 on retinal macrophages in a murine light-induced retinopathy model. Flow cytometry analyses revealed that intake of *L. paracasei* KW3110 significantly increased the ratio of f4/80-, CD11b-, and CD206-positive macrophages in the retina to CD11b-positive cells, 3 days after the light exposure compared with the control mice group (Figure 2A,B). We also evaluated the levels of inflammatory cytokines in retinal macrophages. Intake of *L. paracasei* KW3110 significantly decreased the expression of the inflammatory cytokine TNF-α in retinal macrophages compared with that in the mice group fed a control diet (Figure 2C). In addition, the production of IL-1β (Figure 2D left graph) and RANTES (regulated on activation, normal T cell expressed and secreted) (Figure 2D right graph) inflammatory cytokines, were significantly lower in the mice group fed a diet containing *L. paracasei* KW3110 than that in the control group. These data indicate that intake of *L. paracasei* KW3110 induced M2 macrophages and suppressed the production of inflammatory cytokines evoked by blue light exposure.

### 3.3. Intake of L. paracasei KW3110 Suppresses the Photoreceptor Degeneration Induced by Light Exposure

**Figure 3:**
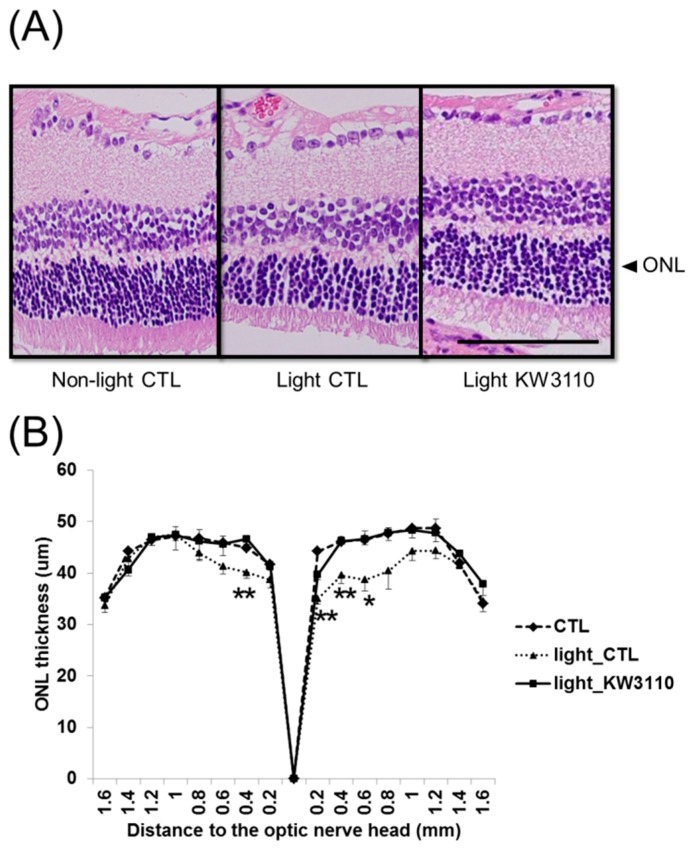
A protective effect of *L. paracasei* KW3110 on light-induced histological retinal changes. (**A**) Hematoxylin and eosin staining of retinal sections. Arrow heads indicate the outer nuclear layer (ONL). Scale bar represents 100 µm. (**B**) ONL thickness was lower in mice fed a control diet than in mice fed a diet with *L. paracasei* KW3110. Values are presented as the mean ± SEM. Significance was assumed if the *p* value was < 0.05. *p < 0.05; **p < 0.01; non-light CTL, no light exposed mice group fed a control diet; light control, light exposed mice group fed a control diet; light KW3110, light exposed mice group fed a diet containing *L. paracasei* KW3110.

Retinal inflammation was previously suggested to be associated with photoreceptor degeneration [10]. To evaluate the effects of *L. paracasei* KW3110 on light-induced retinal degeneration, we compared the ONL thickness containing photoreceptor cells from the optic nerve head to the periphery in the retina. The ONL thickness in the light-exposure mice group fed a control diet was significantly thinner than that in the non-light exposed mice fed a control diet (Figure 3A,B). In contrast, the ONL thickness in the light-exposure mice fed a diet containing *L. paracasei* KW3110 was maintained at the same thickness as in the non-light exposed mice fed a control diet (Figure 3A,B). The ONL thickness in the light-exposure mice group fed a control diet was significantly thinner than that in the light-exposed mice fed a diet containing *L. paracasei* KW3110 (Figure 3A,B and Figure S1). These results indicate that intake of *L. paracasei* KW3110 attenuated photoreceptor degeneration caused by an excessive blue light exposure.

### 3.4. Intake of L. paracasei KW3110 Attenuates the Impairment of Retinal Function

**Figure 4:**
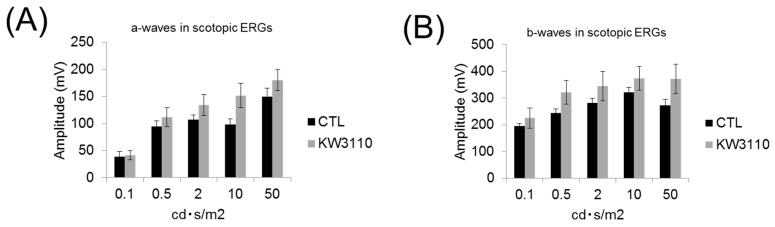

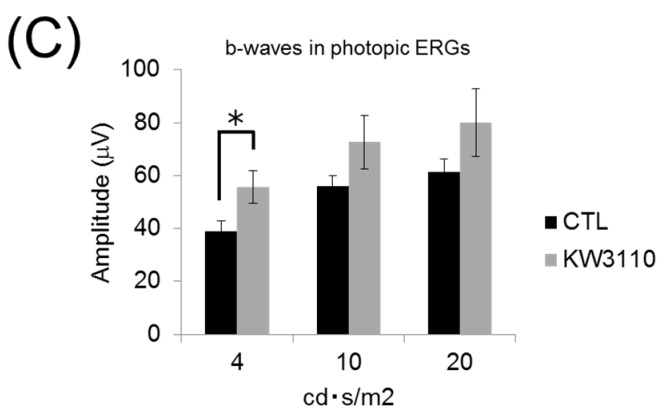
Suppression of visual function impairment by intake of *L. paracasei* KW3110. (**A**) The amplitude of a-waves in the scotopic ERG. (**B**) The amplitude of b-waves in the scotopic ERG. (**C**) The amplitude of b-waves in the photopic ERG. Results are represented as the mean ± SEM. * *p* < 0.05 for the control. CTL, control diet; KW3110, *L. paracasei* KW3110 diet.

To investigate the effects of intake of *L. paracasei* KW3110 on retinal functions, ERG analyses were performed. In the scotopic ERG, the amplitudes of the a- and b-waves tended to be lower in the mice group fed a control diet than in the mice group fed a diet containing *L. paracasei* KW3110 (Figure 4A,B). In addition, the amplitude of the b-wave in the photopic ERG was significantly lower in the mice group fed a control diet than in the mice group fed a diet containing *L. paracasei* KW3110 (Figure 4C). These results suggest that administration of *L. paracasei* KW3110 has a protective effect in both cone and rod photoreceptor functions.

## 4. Discussion

In this study, the in vitro and in vivo experiments suggested that *L. paracasei* KW3110 activated M2 macrophages and induced anti-inflammatory cytokine IL-10 production. We also demonstrated that *L. paracasei* KW3110 had a positive effect on retinal functional restoration in vivo.

In a previous study, ligands for toll-like receptor 2 (TLR2) such as Pam3Cys have been reported to activate mouse dendritic cells and induce IL-10 production through activation of the ERK pathway [28]. In the current study, lipo-teichoic acid (LTA), one of the ligands for TLR2, also slightly activated M2 macrophages and induced IL-10 production. The effect of *L. paracasei* KW3110 on IL-10 production in bone marrow-derived M2 macrophages might have been mediated through the TLR2-dependent ERK pathway since lactic acid bacteria have lipo-teichoic acid. In addition, peptidoglycans in lactic acid bacteria were also reported to increase IL-10 levels via the nucleotide-oligomerization domain receptor 2 (NOD2) pathway [29]. The effects of *L. paracasei* KW3110 on the production of IL-10 might be through the NOD2-dependent pathway at least in part.

In this study, we also showed that *L. paracasei* KW3110 had the potential of activating M2 macrophages and inducing the production of IL-10 in vitro and in vivo (Figure 1A,B). Previously, we reported that orally provided *L. paracasei* KW3110 interacted with the gut immune cells in mice [20]. These results suggested that *L. paracasei* KW3110 could activate the gut immune cells inducing IL-10 production. In a previous report, oral administration of *Pantoea agglomerans*-derived lipopolysaccharide reduced proinflammatory cytokine expression in the blood and reduced the brain Aβ burden and memory impairment [30]. These results suggested that the regulation of cytokine levels, induced by oral administration of food constituents, might have the potential to affect the inflammatory state of the peripheral tissues through systemic blood flow. IL-10 is known as not only one of the M2 macrophage-producing anti-inflammatory cytokines but also one of the factors that induce M2 macrophages [31]. In this study, we demonstrated that intake of *L. paracasei* KW3110 induced CD11b-positive and CD206-positive monocytes which are generally defined as M2 type macrophages in the blue light-exposed retina (Figure 2). Previously, blood-borne macrophages have been reported to integrate into the retina through the optic nerve and the ciliary body in a light-induced retinopathy mouse model [32]. Taken together, *L. paracasei* KW3110 interacts with gut immune cells and might induce M2 macrophages, at least in part, through IL-10 induced from the gut immune cells. Then, those M2 macrophages might be recruited to the retina.

M2 macrophages have been reported to have an anti-inflammatory phenotype when the tissue is damaged [33]. In this study, inflammatory macrophages, i.e., TNF-α-producing macrophages were decreased in the retina of the mice group fed a diet containing *L. paracasei* KW3110 compared with that of mice fed a control diet under the same blue light exposure conditions (Figure 2C). IL-1β and RANTES, which were known as inflammatory phenotype markers in the stressed retina [34,35,36], were also significantly lower in the mice group fed a diet containing *L. paracasei* KW3110 (Figure 2D). In addition, we recently reported that *L. paracasei* KW3110 activated human peripheral blood mononuclear cell- (human-PBMCs) derived M2 macrophages and mitigated VDT load-induced ocular disorders, including eye fatigue, in humans [24]. These results suggested that *L. paracasei* KW3110 induced anti-inflammatory M2 macrophages in the stress conditioned retina.

Intake of *L. paracasei* KW3110 also suppressed light-induced ONL thinning (Figure 3). The ONL is composed of photoreceptor cell bodies and the ONL thickness has been reported to decrease in response to light-induced photoreceptor loss [37]. Although further studies, including analyses of apoptotic cell death of photoreceptors, are needed, intake of *L. paracasei* KW3110 might attenuate photoreceptor loss. We also showed that intake of *L. paracasei* KW3110 could mitigate the impairments of the retinal function evaluated by ERG (Figure 4). The a-wave responses as shown by scotopic ERG indicate rod photoreceptor function and the b-wave responses as shown by scotopic ERG indicate the subsequent responses of photoreceptor function. The b-wave responses as shown by photopic ERG indicate the subsequent response evoked from the cone photoreceptor function [38]. Taken together, it is suggested that intake of *L. paracasei* KW3110 has a protective effect in both cone and rod photoreceptor functions.

Retinal phototoxicity models in small rodents, including a mouse model of light-induced retinopathy, have been widely used in the majority of studies. However, previous studies have demonstrated that light-induced damaged retina showed various morphological patterns in different animal models [39,40,41,42]. In rats and mice, the light-induced damages in the rod photoreceptors have been reported to be more sensitive than in cone photoreceptors [43] while in chickens and pigeons cone photoreceptors have been reported to be damaged first [44]. Therefore, further studies using the larger animals are needed to confirm the preventive effects of *L. paracasei* KW3110 on light-induced inflammation and degeneration in the retina.

## 5. Conclusions

In summary, *L. paracasei* KW3110 induced retinal M2 macrophages in a murine model of light-induced retinopathy. In addition, oral intake of *L. paracasei* KW3110 had a positive effect on retinal morphology and function. These findings suggested that *L. paracasei* KW3110 might have potential as a dietary food supplement to prevent retinal degeneration through regulating inflammation in response to blue-light damage.

## Supplementary Materials

The following are available online at http://www.mdpi.com/2072-6643/10/12/1991/s1. Figure S1: Representative images of H&E staining for retinal sections from the optic nerve head to the most peripheral area.
