# Supplementary material for: Lactobacillus paracasei KW3110 Prevents Blue Light-Induced Inflammation and Degeneration in the Retina
Source: Nutrients. 2018 Dec 15;10(12):1991. doi: 10.3390/nu10121991 (PMC6316514; doi:10.3390/nu10121991)
Supplement: Supplementary file 1 [file nutrients-10-01991-s001.pdf]

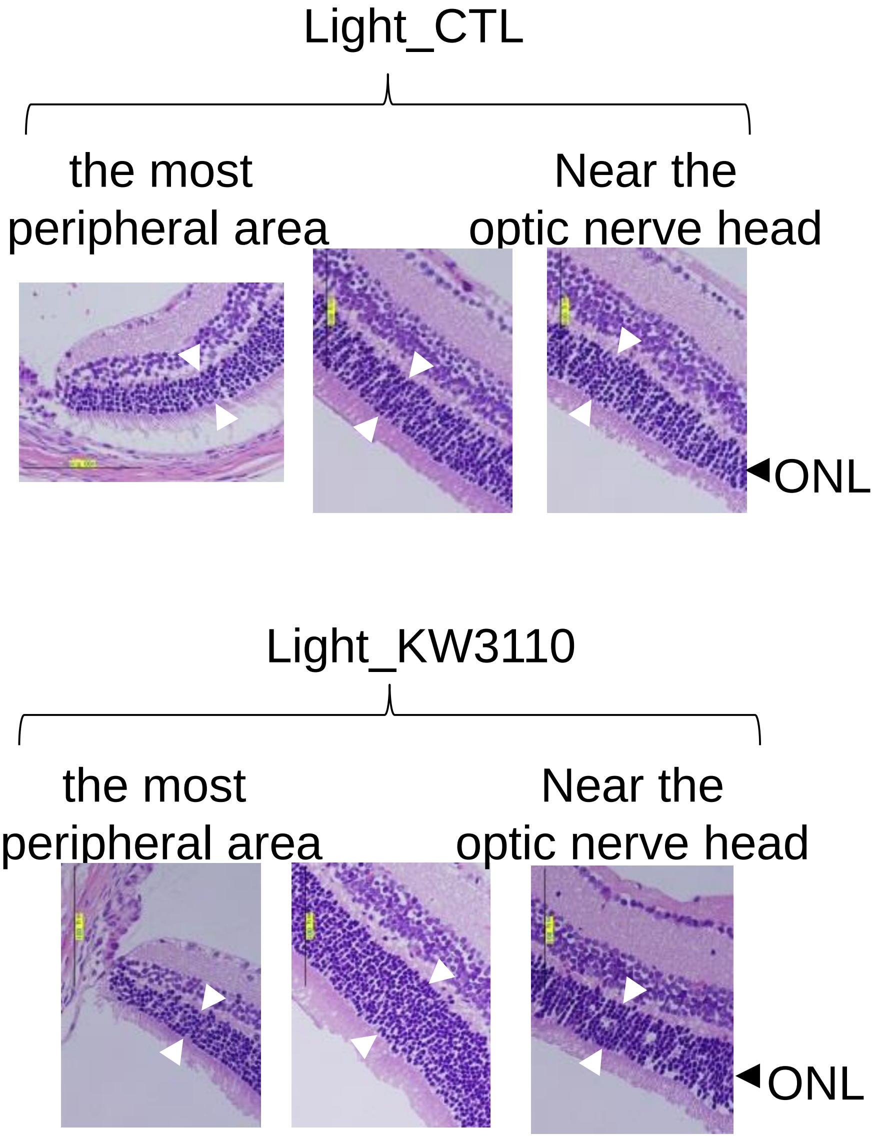

Supplementary Figure 1
